# Survival on railway tracks of *Geranium robertianum*—a glyphosate-tolerant plant

**DOI:** 10.1007/s10646-021-02430-5

**Published:** 2021-06-10

**Authors:** Olga Bemowska-Kałabun, Agnieszka Bogucka, Bogusław Wiłkomirski, Małgorzata Wierzbicka

**Affiliations:** 1 Institute of Environmental Biology, Faculty of Biology, University of Warsaw, I. Miecznikowa 1, 02-096 Warsaw, Poland; 2 Institute of Geography and Environmental Science, The Jan Kochanowski University in Kielce, Świętokrzyska 15, 25-406 Kielce, Poland

**Keywords:** *Geranium robertianum* L., Herbicide, Glyphosate, AMPA, Railway tracks

## Abstract

*Geranium robertianum* is a herbaceous plant that prefers shady and fertile forest habitats. However, it also occurs on railway tracks, where there are difficult conditions for plant growth and regular herbicide spraying (in high concentrations, twice a year). One of the most commonly used herbicides in railway areas is glyphosate. The effect of the glyphosate on the *G. robertianum* plants found on railway tracks and in nearby forests in north-eastern Poland was checked. The aim of the study was to explain how *G. robertianum* can survive on railway tracks despite spraying with the glyphosate. Increased tolerance to the glyphosate of the *G. robertianum* plants from track populations was demonstrated compared to the plants from forest populations that had not previously been in contact with the herbicide. After 35 days after treatment with the herbicide, 75% of the plants from the observed forest populations withered, while only 38% did from the track populations. Ultrastructure of plant leaf cells from forest populations was strongly disturbed, which was not observed in plants from track populations. It was also shown that plants from track populations accumulated more glyphosate and AMPA in their tissues than plants from forest populations. The obtained results indicate that long-term use of herbicides may cause formation of biotypes of plants resistant to a given herbicide. This fact explains the possibility of *G. robertianum* occurring on railway tracks, despite spraying with the glyphosate. It is also a manifestation of microevolutionary processes.

## Introduction

Railway tracks are a very good object to assess effects of the long-time use of herbicides. This is because the doses of herbicides used on railway tracks are high compared to those used in agricultural areas, and are used regularly for several dozen years (Schweinsberg et al. 1999; Bӧrjesson and Torstensson 2000; Torstensson 2001; Torstensson et al. 2005; Burkhardt et al. 2008; Adamczewski et al. 2011; Wierzbicka et al. 2015). This gives an opportunity to check whether the plants acquire resistance to the herbicide, and thus what the environmental effects of its excessive use are. For example, in Germany 90 percent of around 19,000 tonnes of all herbicides in 1990 was used in agriculture on an area of 120,000 km^2^. It was thus used 1.4 tonne of herbicides per hectare. For comparison, in the same year, about 200–250 tonnes of herbicides were used on railway tracks in Germany on an area of 245 km^2^, which corresponds to 8–10 tonnes per hectare. Thus, the amount of herbicides used on railway tracks was six times higher than that used in agriculture (Schweinsberg et al. 1999). Railway tracks in Poland are also places where significant and regular herbicide spraying is carried out (Adamczewski et al. 2011; Mętrak et al. 2015).

One of the more commonly used herbicides in railway areas is Roundup, whose an active substance is glyphosate (N-(phosphonomethyl)glycine). It is a foliar, non-selective herbicide with systemic effects. Glyphosate is present in Roundup as the isopropylamine salt. Spraying with the glyphosate is used on railway embankments to remove plants. Spraying is carried out twice a year. High doses of herbicide are used (Baylis 2000; Torstensson 2001; Kudsk and Streibig 2003; Torstensson et al. 2005; Das and Mondal 2014). The dose recommended by the Roundup’s manufacturer on railway tracks is 7 l/ha, i.e., 3.5 l/km of a track, with the 5-m wide zone of removing weeds. Spraying is carried out during the period of intensive growth of green plants. Doses and dates of spraying should also be selected in relation to the plants occurring there (Roundup Label 360 SL 2009).

The environmental consequence of the intensive use of herbicides may be, for example: the destruction of organisms that were not a target of the herbicide, affection of plants reproduction, or the development of resistance by these organisms to the herbicide if the dose was not lethal for them. The possibility that new plant form resistant to herbicide will appear, may be a side effect of high herbicide use on railway areas, with great significance and unfavorable influence on environment, as well as for human economic activities. Herbicide-resistant plants may become dominant in the area over time, displacing herbicide-sensitive species present there. This will pose a threat to the biodiversity of organisms in the vicinity of the site where the herbicides are applied. The consequence of the plant resistance from human point of view, will be for example a reduction in the level of effectiveness of the herbicide, which may ultimately limit the usefulness of the product or its chemical group, which is associated with an increase in economic costs (Adamczewski et al. 2011; Strandberg et al. 2012; Boutin et al. 2014; Rao 2014; EPPO Standard PP 1/213(4) 2015; Buddenhagen et al. 2019). The presence of resistant’s plants can also weaken the structure of railway embankments. The examples above show how important it is to study the emergence of new herbicide-resistant species.

Among the species found on railway embankments in north-eastern Poland, *Geranium robertianum* L. (Geraniaceae Juss.) distinguished itself, as it occurred abundantly on railway tracks. *G. robertianum* is a herbaceous plant with an annual or biennial development cycle, although some authors suggest that this species may also be perennial. *G. robertianum* is classified according to the Raunkiaer system as a hemicryptophyte or less frequently by some authors as a terophyte (Falinska and Piroznikov 1983; Grime et al. 1988; Bertin 2001; Tofts 2004; Vandelook and Van Assche 2010; Galera et al. 2011, 2012). These plants prefer shady and fertile forest habitats with a high content of phosphorus and nitrogen in the soil. However, opposite conditions prevail on railway tracks—strong insolation and very nutrient-poor substrate (crushed stone mixed with river sand), and extensive use of herbicides (Falinska and Piroznikov 1983; Tofts 2004; Buriánek et al. 2013; Májeková et al. 2014; Wierzbicka et al. 2014). It is interesting, therefore, as a result of which processes this ecological niche is colonized by the *G. robertianum* plants.

The aim of the research described in this paper was to explain how *G. robertianum* can survive on railway tracks despite strong spraying with the glyphosate. It was checked whether track populations of *G. robertianum* near Białystok have increased tolerance to the glyphosate compared to forest populations of this species near Białystok.

## Materials and methods

### Plant cultivation

The object of research was *Geranium robertianum* L. (Geraniaceae Juss.). Seeds from the studied populations of *G. robertianum* came from the railway line No. 37 Białystok – Zubki Białostockie (track populations) and from direct neighborhood of this railway line (forest populations), in north-eastern Poland. Seeds of selected populations were collected in the years 2008, 2014–2016 (Table 1). Plants from F1 generation were tested.

**Table 1:** Locations of seed collection; from these seeds the examined railway track and forest populations of *G. robertianum* were cultivated (in the order of their occurrence from west to east, starting from Białystok)

No.	Population	GPS coordinates	Population type	Description of places where *G. robertianum* occurs
1.	Białystok Fabryczny Station	N53°08’18.3”; E023°11'23.0”	Railway track	Big railway station Białystok Fabryczny; several tracks. Around the station: settlements, trees and scrap yard. Seed collection year: 2014
2.	Zajezierce	N53°05’38.9”; E023°21'50.7”	Forest	Deciduous-coniferous forest behind the village of Zajezierce; by the forest road. Seed collection year: 2014
3.	Waliły-Station (2008)	N53°06’28.7”; E023°39’00.3”	Railway track	Point located under the platform (ramp) of the Waliły-Station. Plants grown from these seeds previously studied by Wierzbicka et al. (2014). Seeds collection year: 2008
4.	Waliły-Station (2015/2016)	N53°06’28.7”; E023°39’00.3”	Railway track	Point located under the platform (ramp) of the Waliły-Station. From this point (under the ramp) seeds collected in 2008 came, the population described by Wierzbicka et al. (2014). Seeds collection year: 2015, 2016
5.	Gródek	N53°06’04.2”; E023°38'41.5”	Forest	Deciduous forest; edge of the forest, by the road. Near the village Gródek and Waliły-Station. Damp, fertile habitat. Seed collection year: 2014
6.	Zubki Białostockie Station	N53°05’00.1”; E023°47’43.8”	Railway track	The end station of railway line No. 37 – Zubki Białostockie, several tracks. The station is currently closed. The tracks are heavily overgrown with grasses. Around the station are coniferous forests. Seed collection year: 2014

Seeds from five populations (Table 1) were sown in Petri dishes (300 seeds were sown from each population, and each of 50 seeds were placed in separate Petri dish on three layers of filter paper), and placed in a phytotron chamber. Seedlings were grown in a phytotron for 20–30 days (day: 22 °C for 16 h and night: 20 °C for 8 h). Then the seedlings were transplanted into pots with garden soil (Universal substrate, SUBSTRAL). In a greenhouse plants grew in a natural photoperiod at a temperature of +25 ± 5°C and humidity of 50 ± 15%. The plants were regularly watered and fertilized with Florovit.

Finally, 175 plants were obtained in total from all populations. Over a dozen plants for each population (from 10 to about 40 plants, depending on the number of plants obtained in cultivation) were tested in all experiments. Adult *G. robertianum* plants at middle growth vegetative stage (characterized by plants with ten or more leaves, but before the generative phase) were used for the all experiments. A detailed description of the conducted experiments can be found below.

### The use of the herbicide—experimental scheme

A single dose of the herbicide was used to spray the plants. The recommended dose on railway tracks was selected (the dose was calculated so that spraying on a single plant could be used): 720 mg/l glyphosate, 25 ml solution per a plant (18,000 µg glyphosate). The herbicide Roundup Ultra 170 SL was used to spray the plants. The content of the active substance, glyphosate, in this preparation is 170 g in 1 l of the agent (Safety data sheet Roundup Ultra 170 SL 2015). Based on the Roundup herbicide and distilled water, aqueous solutions with the appropriate glyphosate concentration were prepared by volume. To carry out spraying with the herbicide, the Hozelock Plus 5 l pressure sprayer was used, which allowed spraying in a volume of 25 ml of solution per a plant (spot application). Non-herbicide-treated plants were the control in all experiments. The method of plants herbicide treatment was in compliance with the guidelines from the EPPO Standard PP 1/239(2) (2012).

### Macroscopic and TEM observations

After spraying plants at middle growth stage with the herbicide the visual assessment (EPPO Standard PP 1/181(4) 2012; EPPO Standard PP 1/135(4) 2014) has been carried out for 35 days. Macroscopic observations were made for plants of forest populations from: Zajezierce and Gródek, as well as for plants of track populations from: Białystok Fabryczny Station, Waliły-Station (2008), Waliły-Station (2015/2016) and Zubki Białostockie Station. In total 175 plants, over a dozen plants for each population, were tested. The control and herbicide-treated plants were tested in each population. Observations were made of such parameters as: a number of leaves, leaf color, presence of chlorosis and necrosis, condition of the plants, presence of withered or withering leaves. As a part of the study, macroscopic observations of the whole *G. robertianum* plants and their leaves, leaf observations under a binocular equipped with a digital camera and an image analysis system were made (NIS-Elements BR 3.0, Nikon). Chlorosis observations were made on the 70 days after treatment (DAT) with glyphosate, while the observations of the remaining parameters were carried out until the 35 DAT.

For observation in the transmission electron microscope, the plants at middle growth stage of the track populations from Waliły-Station (2015/2016) and Białystok Fabryczny Station, and of the forest populations from Zajezierce, treated with the glyphosate, were used. The control was unsprayed plants of the forest population from Zajezierce. For each variant two *G. robertianum* plants from the relevant population were tested. Observations of semi-thin specimens were made in the light microscope (Nikon EFD-3). Observations of leaf cells ultrastructure were performed in control variants and in trials after the herbicide spraying in the transmission electron microscope JEM 1400 (JEOL Co., Japan 2008). A conventional technique with glutaraldehyde and OsO_4_ was used to prepare the material for TEM studies. Material fixation was carried out at room temperature (Antosiewicz and Wierzbicka 1999). Leaf fragments 2 × 3 mm, of the *G. robertianum* plants were collected for fixation, cut from the middle part of the leaf blade.

### Glyphosate and AMPA concentration in plants after herbicide spraying

The content of glyphosate and its main degradation product – AMPA (aminomethylphosphonic acid) was tested in aboveground parts after spraying with glyphosate the *G. robertianum* plants of the track populations: Białystok Fabryczny Station (*N* = 16), Waliły-Station (2008) (*N* = 23), Waliły-Station (2015/2016) (*N* = 9), Zubki Białostockie Station (*N* = 6), and the forest populations: Zajezierce (*N* = 26), Gródek (*N* = 5). The day after the herbicide spraying, the number of leaves on each *G. robertianum* plant from each population was counted. Then, the aboveground parts of the plants were cut off and weighed. Next, the plants of *G. robertianum* from individual populations were divided into samples (combined of 5–6 plants in a sample). In total, 10 samples from the track populations and 6 samples from the forest populations were obtained. The samples were subjected to chemical analyses in terms of the content of glyphosate and its main degradation product, AMPA. After homogenization in a mill, 4 g of material were weighed from each sample and 10 ml H_2_O and 10 ml MeOH were added to obtain an extract of 0.2 g in 1 ml. Glyphosate and AMPA were determined by LC/MS/MS method, based on the Anastassiades et al. (2019) protocol. Tests for glyphosate and AMPA were carried out in the accredited laboratory of Hamilton UO-Technologia Sp. z o.o. (J.S. Hamilton Poland).

### Statistical analyses

Statistical analyses were performed using the STATISTICA program, version 13.1. (StatSoft, Inc. 2014). To compare plants in many groups the non-parametric Kruskal–Wallis test was used for many independent samples, while when comparing two groups the Mann–Whitney *U* test was used for two independent samples (significance level *α* = 0.01). The charts show arithmetic means with standard deviations. Statistical analysis was performed based on the EPPO Standard PP 1/152(4) (2012).

## Results

### Comparison of plant reactions to herbicide between track and forest populations

#### Macroscopic observations

**Fig. 1:**
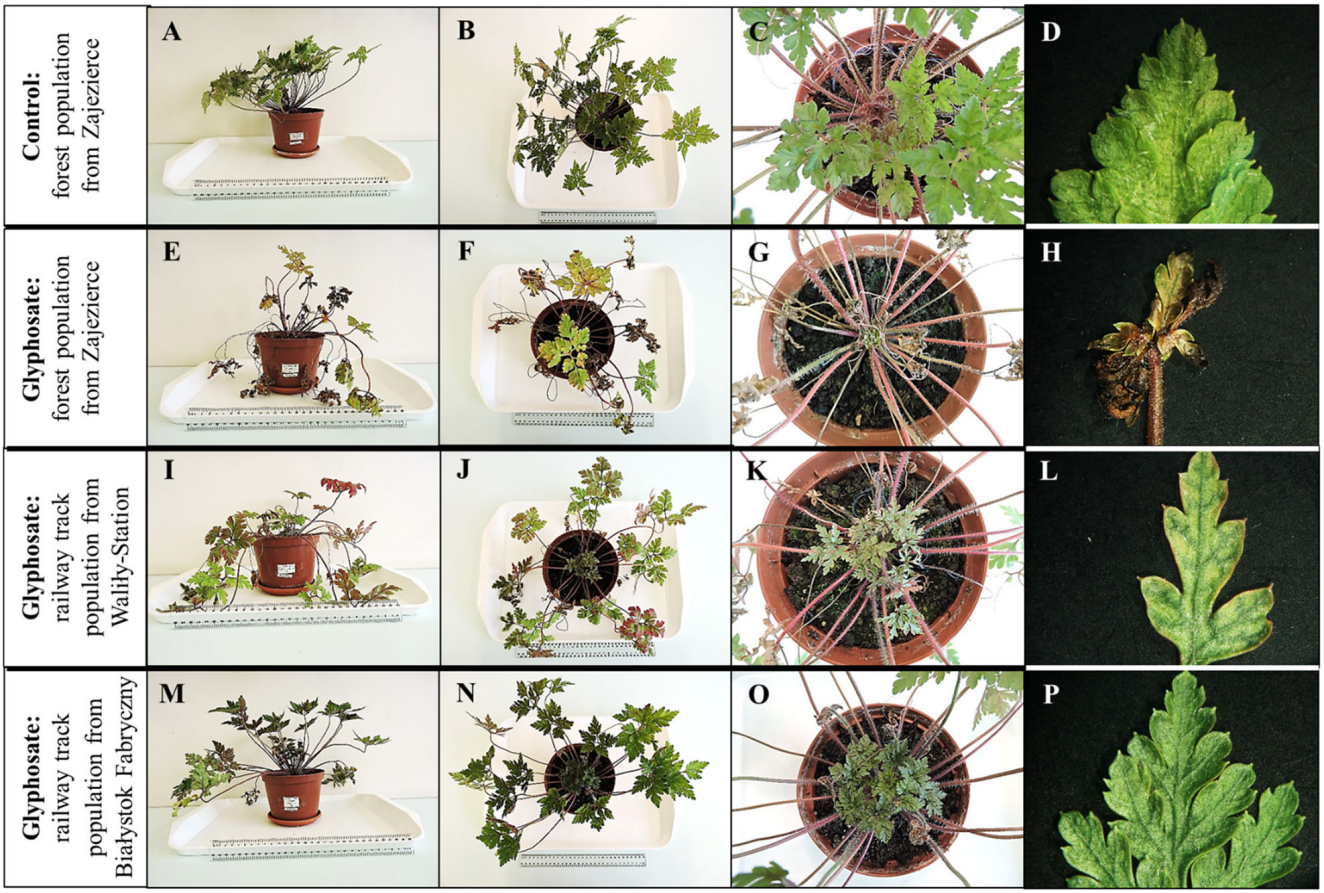
*G. robertianum* plant and leaf blades of its youngest leaves from: control—forest population from Zajezierce (**A**–**D**). *G. robertianum* plants and leaf blades of their youngest leaves 2 months after treatment with herbicide (720 mg/l glyphosate, 25 ml solution per plant), from: forest population from Zajezierce (**E**–**H**), railway track population from Waliły-Station (2015/2016) (**I**–**L**) and from Białystok Fabryczny (**M**–**P**). Camera (**A**–**C**, **E**–**G**, **I**–**K**, **M**–**O**) and binocular (**D**, **H**, **I**, **P**), magnification 1×

**Fig. 2:**
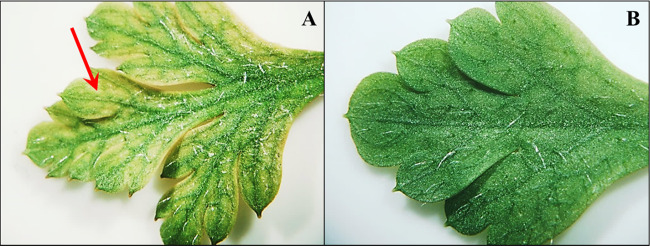
Appearance of young leaf of *G. robertianum* plant from railway track population 16 (**A**) and 21 DAT (**B**). The red arrow indicates chlorosis between leaf veins. Binocular, magnification 1×. On leaves of plants from railway track populations, gradual leaf regeneration was observed

**Fig. 3:**
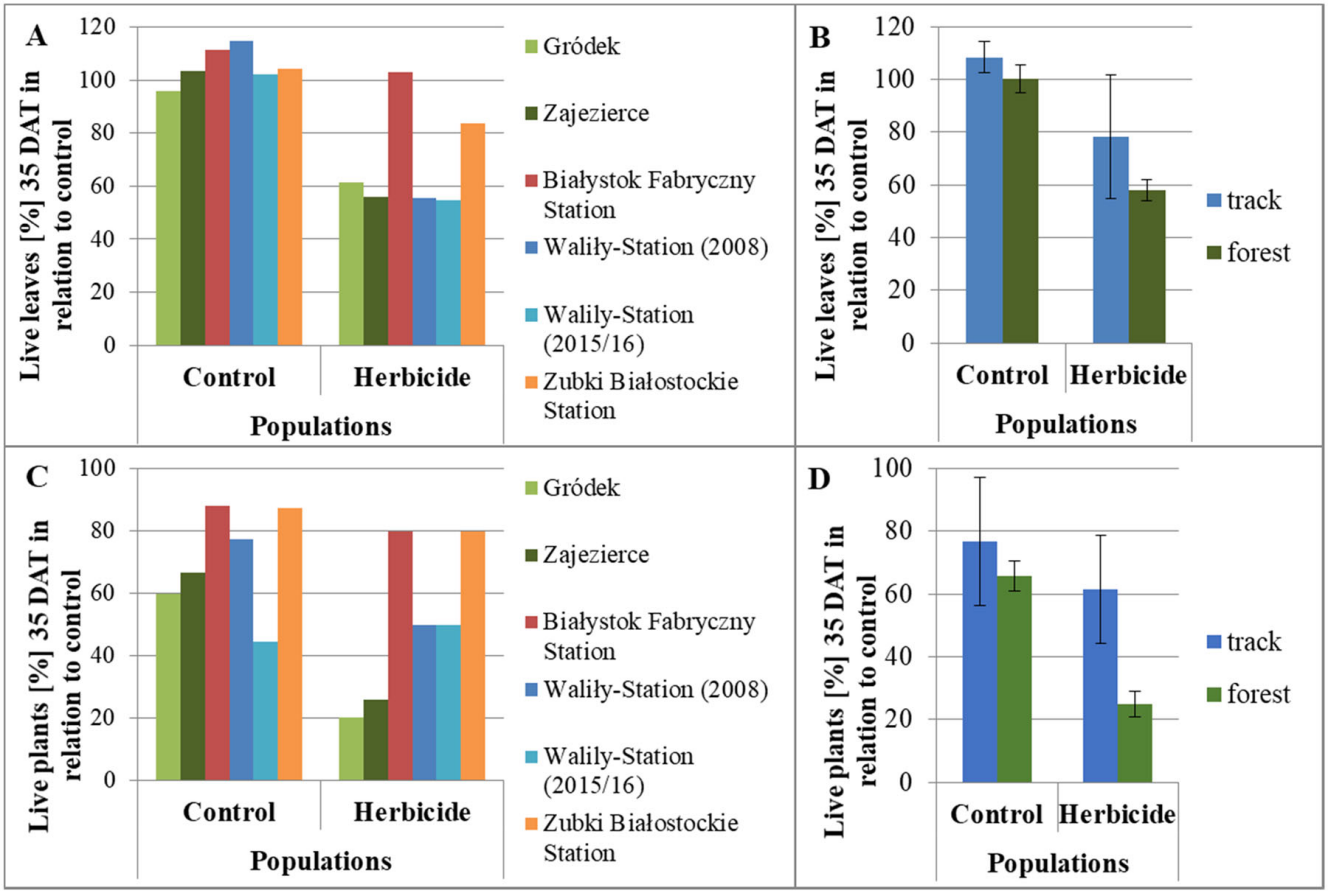
Live leaves on *G. robertianum* plants [%] 35 days after treatment with herbicide relative to control, from individual railway track and forest populations (**A**), and in all railway track and forest populations (**B**). Live plants of *G. robertianum* [%] 35 days after treatment with herbicide relative to control, from individual railway track and forest populations (**C**), and in all railway track and forest populations (**D**). Number of plants tested in control: the track populations—Białystok Fabryczny Station (*N* = 17), Waliły-Station (2008) (*N* = 22), Waliły-Station (2015/2016) (*N* = 9), Zubki Białostockie Station (*N* = 8), and the forest populations—Zajezierce (*N* = 30), Gródek (*N* = 5). Number of plants tested after spraying with herbicide: the track populations—Białystok Fabryczny Station (*N* = 15), Waliły-Station (2008) (*N* = 24), Waliły-Station (2015/2016) (*N* = 8), Zubki Białostockie Station (*N* = 5), and the forest populations—Zajezierce (*N* = 27), Gródek (*N* = 5)

The *G. robertianum* plants from the control variants were in good condition throughout the observation period, and no changes were observed on their youngest leaves (Fig. 1). In the *G. robertianum* plants from both track and forest populations, about 14 days after treatment (DAT) with glyphosate, chlorosis appeared between the veins on the youngest leaves (Fig. 1H, L). Further macroscopic observations showed that both the youngest and older leaves of plants from the forest populations of Zajezierce and Gródek withered within a month of spraying with the herbicide. The percentage of live leaves on plants from the forest populations was about half lower than in the control 35 DAT (Figs. 1E–H and 2). In the youngest leaves of plants from Waliły-Station and Zubki Białostockie Station both chlorosis and slightly changed shape of leaf blades were observed after about a month (Fig. 1I–L). The least damaged youngest leaves were observed in plants from Białystok Fabryczny Station—chlorosis was low or absent on these leaves, and unchanged shape of leaf blades was observed (Fig. 1M–P). On the youngest leaves of the *G. robertianum* plants from the track populations, chlorosis after the herbicide spraying began to disappear gradually, until complete regeneration of leaves in the case of the track population from Białystok Fabryczny or partial regeneration of leaves in the case of other track populations. Significant disappearance of chlorosis was visible about 30–40 DAT, while almost complete 60–70 DAT (Fig. 2). No such phenomenon was observed in plants from the forest populations. The percentage of live leaves on plants from the track populations was only about a quarter lower than in the control 35 DAT. More live leaves were observed in the track rather than forest populations 35 DAT, especially in the populations from the Białystok Fabryczny and Zubki Białostockie Stations (Fig. 3A, B).

In the case of the forest populations, most plants withered 35 DAT. The lowest tolerance to the herbicide was demonstrated in plants from the Gródek forest population. Plants from the Zajezierce forest population were characterized by slightly higher herbicide tolerance than plants from the Gródek population. A total of 75% of the plants from both observed forest populations withered 35 DAT (Fig. 3C, D). In the case of plants from the track populations of Białystok Fabryczny, Waliły-Station and Zubki Białostockie, after a month of the herbicide spraying, the vast majority of plants remained alive. 35 DAT, only about 38% of plants from the tested track populations withered, which is almost twice less than in the case of plants from the forest populations. For plants of the track populations, tolerance to a single dose of the glyphosate used on railway tracks was varied. Plants from the Zubki Białostockie and Białystok Fabryczny stations showed the highest tolerance to the herbicide, while average tolerance was shown by plants of both tested track populations from Waliły-Station (Fig. 3C).

In summary, after spraying with the glyphosate, both whole plants and the youngest leaves of plants from the track populations were in better condition, compared to plants from the forest populations (Figs. 1–3).

#### Observations in the light microscope

The control plants from Zajezierce were characterized by a typical structure of a leaf blade. No damaged cells were observed here. However, after spraying with glyphosate in leaf blades of the *G. robertianum* plants from Zajezierce there were observed i.a.: shrunken palisade and spongy mesophyll cells, dark deposits in both mesophyll and epidermal cells and accumulation of characteristic gray structures in numerous cells.

In the leaves of plants from the track populations (Waliły-Station (2015/2016) and Białystok Fabryczny), after spraying with herbicide no shrunken mesophyll cells were observed. However, there were observed dark deposits in the cells of both mesophylls and epidermis and accumulation of characteristic gray, flocculent structures in some cells. There were more such cells with dark deposits in the track variants than in the variant with the forest population from Zajezierce after the herbicide spraying. Especially a lot of cells with dark deposits were in the variant from the Waliły-Station population (2015/2016).

These observations testify to differences in tolerance to the glyphosate between plants from railway tracks (higher tolerance—greater accumulation of dark deposits in cells without damage to cells, lack of shrunken cells in leaf blades) and plants from the forest (lower tolerance—shrunken cells of both mesophylls).

#### Observations in the transmission electron microscope

**Fig. 4:**
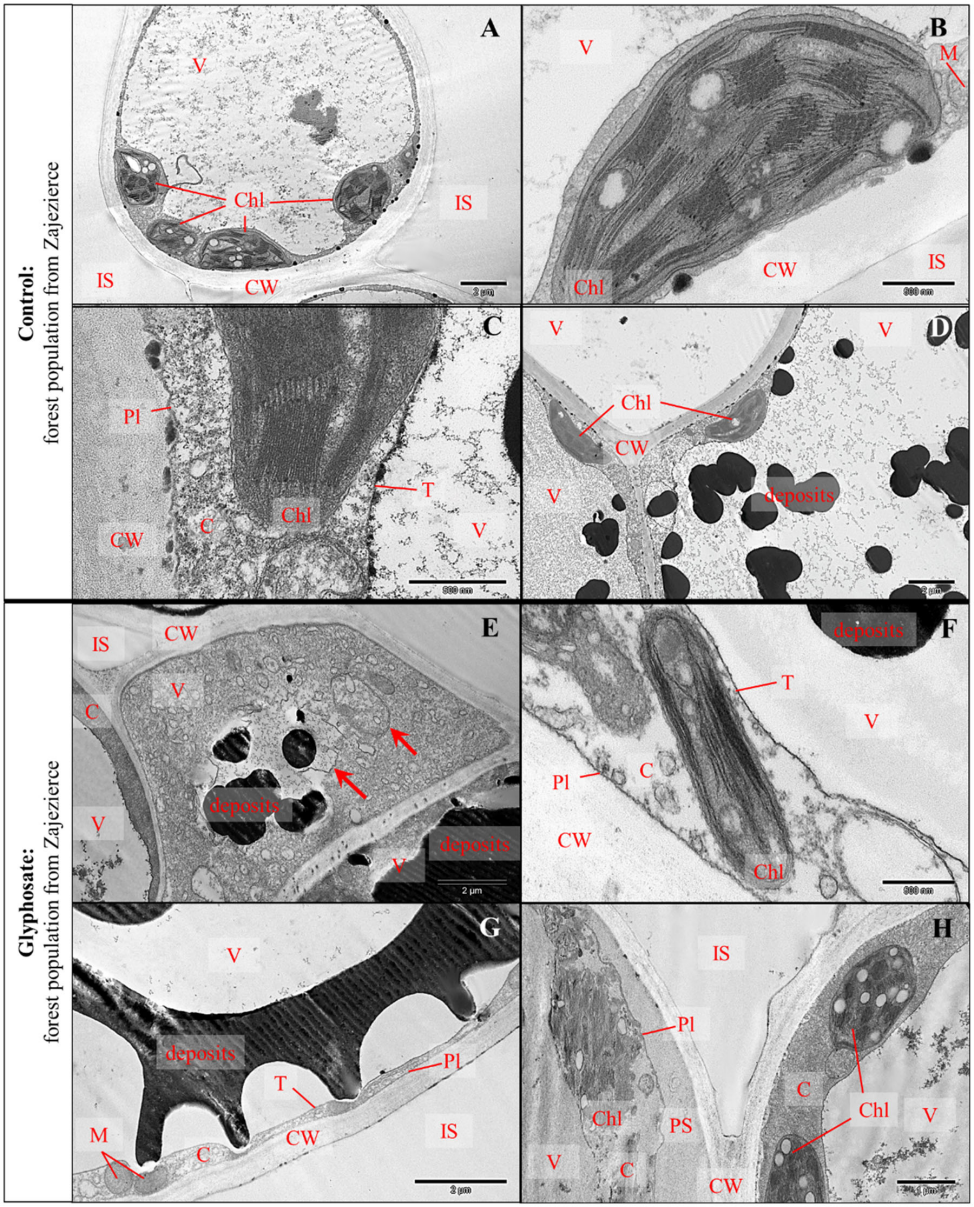
TEM, cells ultrastructure on cross-sections through leaf blades of *G. robertianum* plants from Zajezierce forest population in the control variant (**A**–**D**): magnification 5000×: sponge mesophyll, typical ultrastructure, chloroplasts with and without starch grains (**A**); magnification 60,000×: sponge mesophyll, chloroplast without starch grains, typical ultrastructure (**B**); magnification 80,000×: sponge mesophyll, chloroplast fragment and cytoplasm, typical ultrastructure (**C**); magnification 10,000×: palisade mesophyll, chloroplasts without starch grains, electron-dense deposits in vacuoles and gray flocculent content (**D**). TEM, cell ultrastructure of cross-sections through leaf blades of *G. robertianum* plants from Zajezierce forest population, a month after spraying with herbicide (720 mg/l glyphosate, 25 ml solution per plant) (**E**–**H**): magnification 15,000×: lower epidermis cells, vacuoles with electron-dense deposits, flocculent contents and additional compartments (**E**); magnification 60,000×: palisade mesophyll, flattened chloroplast with disturbed ultrastructure, disturbed cytoplasm, electron-dense deposits in vacuole (**F**); magnification 20,000×: palisade mesophyll, electro-dense deposits in vacuole with characteristic shape (**G**); magnification 25,000×: palisade mesophyll, disintegration of chloroplast with disturbed ultrastructure, disturbed cytoplasm, visible periplasmic space, plasmolysis, chloroplast without starch grains (**H**). Markings on electronograms: CW cell wall, C cytoplasm, V vacuole, M mitochondrium, Chl chloroplast, IS intercellular space, T tonoplast, Pl cell membrane, PS periplasmic space, by plasmolysis; electron-dense deposits. The arrows indicate additional compartments in vacuole

The ultrastructure of the control plant leaf cells was unchanged. Chloroplasts were both with and without starch grains and had typical ultrastructure (Fig. 4A–C). Flocculent content and few electron-dense deposits were observed in cell vacuoles (Fig. 4D). No damaged cells were observed.

After spraying with the glyphosate in the *G. robertianum* plants of the Zajezierce forest population, strong changes in cell ultrastructure were observed. Among the changes there were: formation of numerous additional membrane compartments in vacuoles and electron-dense deposits in various cellular compartments with characteristic shapes, occupying a significant part of the vacuoles (Fig. 4E, G). Chloroplasts with damaged ultrastructure (both with and without starch grains) were observed in the palisade and spongy mesophyll cells as well as in the upper and lower epidermis. Cytoplasm with changed structure was also observed here. Periplasmic spaces near the cell walls resulting from plasmolysis were also visible (Fig. 4F, H).

**Fig. 5:**
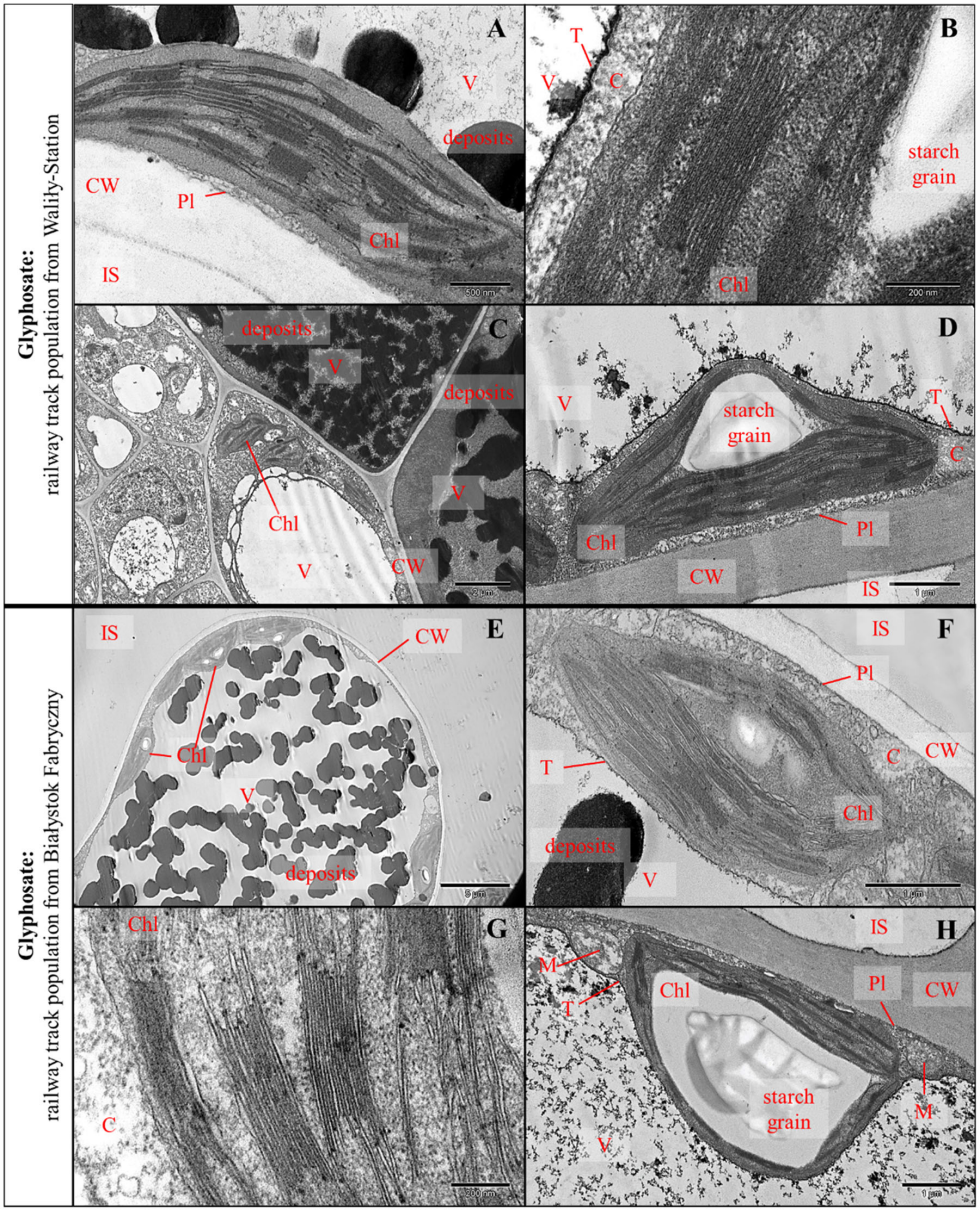
TEM, cell ultrastructure of cross-sections through leaf blades of *G. robertianum* plants from Waliły-Station (2015/2016) (**A**–**D**) and Białystok Fabryczny railway track populations (**E**–**H**), a month after spraying with herbicide (720 mg/l glyphosate, 25 ml solution per plant): magnification 50,000×: palisade mesophyll, chloroplast without starch grains with typical ultrastructure, vacuole with electron-dense deposits and flocculent content (**A**); magnification 150,000×: sponge mesophyll, fragment of chloroplast with typical ultrastructure with starch grain, cytoplasm with typical structure (**B**); magnification 12,000×: sponge mesophyll near conductive bundle, electron-dense deposits and flocculent content in vacuoles (**C**); magnification 30,000×: sponge mesophyll, chloroplast with large starch grain with typical ultrastructure (**D**); magnification 6000×: sponge mesophyll, chloroplasts with and without starch grains, vacuole with electron-dense deposits (**E**); magnification 40,000×: sponge mesophyll, chloroplast with small starch grain, electron-dense deposits in vacuole (**F**); magnification 120,000×: sponge mesophyll near conductive bundle, a fragment of chloroplast with typical ultrastructure, cytoplasm with typical structure (**G**); magnification 25,000×: sponge mesophyll, chloroplast with very large starch grains, flocculent content in vacuoles (**H**). Markings on electronograms: CW cell wall, C cytoplasm, V vacuole, M mitochondrium, Chl chloroplast, IS intercellular space, T tonoplast, Pl cell membrane; starch grains; electron-dense deposits

The *G. robertianum* plants from the track populations were also treated with the glyphosate. In plant leaf cells of the track populations from Waliły-Station and Białystok Fabryczny there were not changed cells or damaged chloroplasts (Fig. 5A, B, E–G). Only numerous electron-dense deposits occupying almost the entire vacuoles were present. Deposits were observed in the palisade and spongy mesophyll cells as well as the upper and lower epidermis, with the largest number in the spongy mesophyll and lower epidermis (Fig. 5C, E). There were more electron-dense deposits in leaf cells of plants from Waliły than from Białystok. In the leaf cells of plants from the railway track populations there were more deposits in vacuoles than in the control plants and those ones of the forest population from Zajezierce, after the herbicide spraying. Chloroplasts with and without starch grains were observed in plant cells from the railway track populations (Fig. 5A, D, F–H). In plant cells from Białystok starch grains in chloroplasts were particularly large, definitely larger than in the control plants.

#### Glyphosate and AMPA concentration in *G. robertianum* plants

**Fig. 6:**
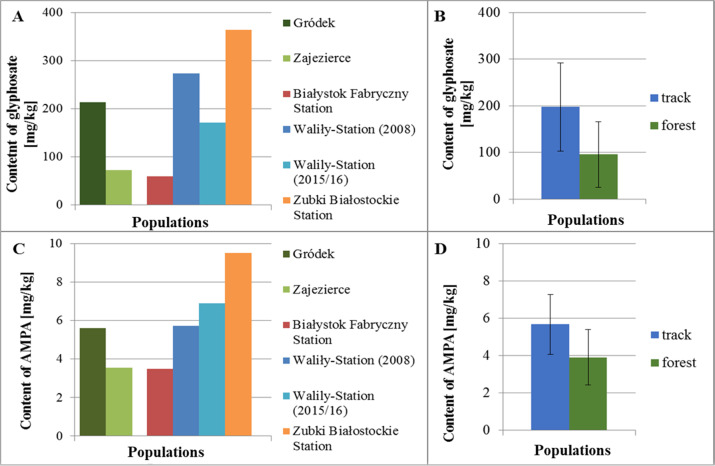
The content of glyphosate (**A**) and AMPA (**C**) [mg/kg] in the tested plant samples from individual railway track populations and forest populations of *G. robertianum* (for chemical studies, samples consisted of mixed individuals from a given population, therefore for each population one result was obtained), as well as the content of glyphosate (**B**) and AMPA (**D**) together in all railway track populations and all forest populations; the plants were taken for testing the day after treatment with herbicide (**A**–**D**). The size of the samples: the track populations—Białystok Fabryczny Station (*N* = 16), Waliły-Station (2008) (*N* = 23), Waliły-Station (2015/2016) (*N* = 9), Zubki Białostockie Station (*N* = 6), and the forest populations—Zajezierce (*N* = 26), Gródek (*N* = 5)

It was checked what dose of the glyphosate and AMPA the *G. robertianum* plants from the track and forest populations accumulate. Both the glyphosate and AMPA content in the studied populations was quite diverse. The highest content of glyphosate and AMPA was found in plants of the railway track population from Zubki Białostockie and the lowest in plants of the railway track population from Białystok Fabryczny. In most cases, higher content of both compounds occurred in plants from the railway track populations rather than forest populations. The average glyphosate content in plants from the railway track populations was 197.72 mg/kg, and AMPA 5.67 mg/kg, whereas the average content of glyphosate in plants from the forest populations was 95.52 mg/kg, and AMPA 3.90 mg/kg (Fig. 6A–D).

**Fig. 7:**
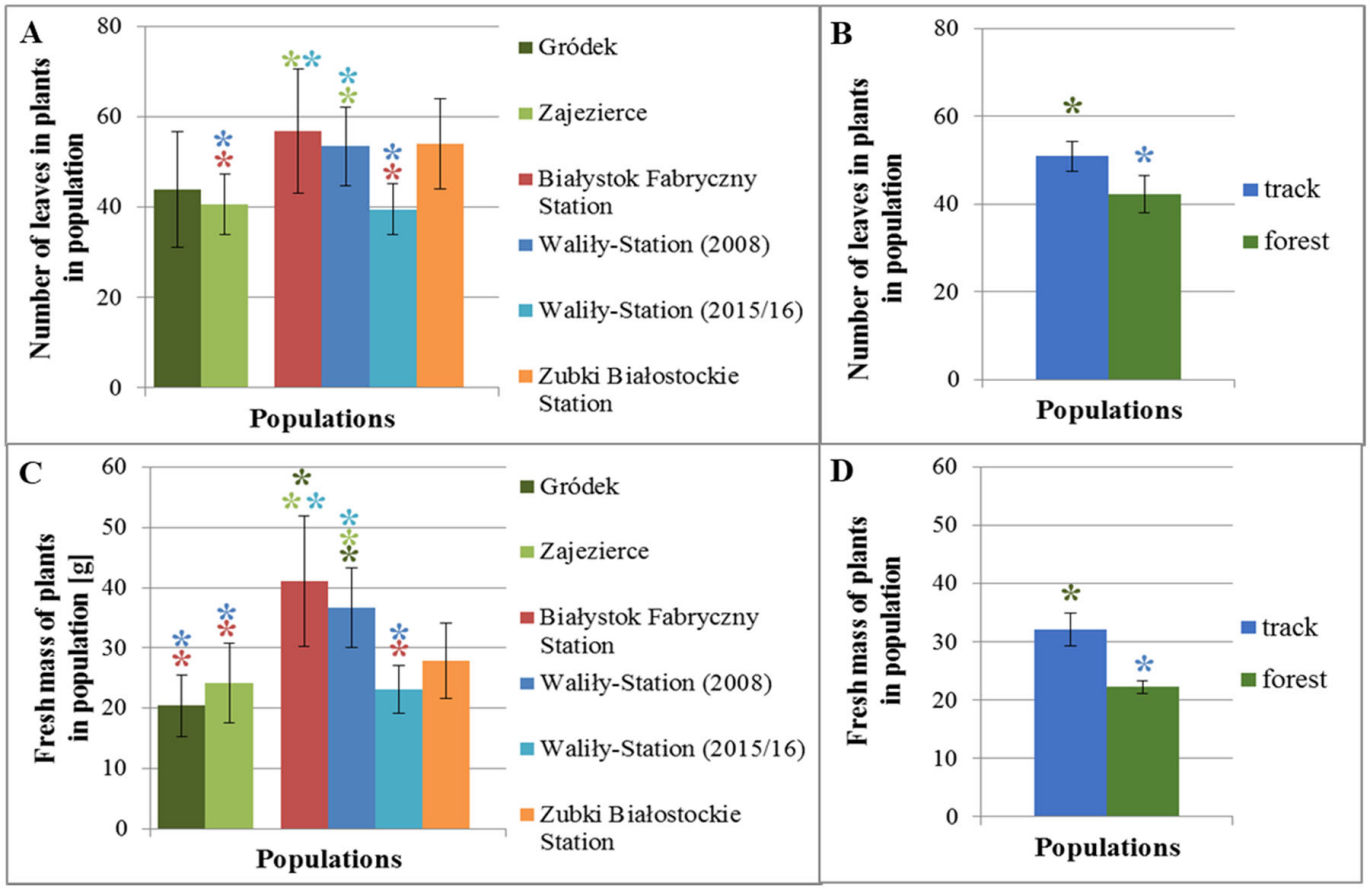
The number of leaves in rosettes in *G. robertianum* plants from individual railway track and forest populations (**A**) and railway track and forest populations together (**B**), used for chemical research. Rosettes fresh weight [g] in *G. robertianum* plants from individual railway track and forest populations (**C**) and railway track and forest populations together (**D**), used for chemical research. The measurement was made the day after treatment with herbicide (**A**–**D**). Asterisks indicate statistically significant differences between individual railway track and forest populations (Kruskal–Wallis test; significance level *α* = 0.01) (**A**, **C**) and for railway track and forest populations together (Mann–Whitney *U* test; level of significance *α* = 0.01) (**B**, **D**). The size of the samples: the track populations—Białystok Fabryczny Station (*N* = 16), Waliły-Station (2008) (*N* = 23), Waliły-Station (2015/2016) (*N* = 9), Zubki Białostockie Station (*N* = 6), and the forest populations—Zajezierce (*N* = 26), Gródek (*N* = 5)

The glyphosate and AMPA concentration may have been dependent on plants’ size. Therefore, to relate the obtained results of the glyphosate and AMPA content to the plants’ size, leaves of the *G. robertianum* plants were counted and fresh weight of their rosettes was measured. Among the studied populations, plants from the Białystok Fabryczny population had the largest number of leaves and plants of the populations from Waliły-Station (2015/2016) and Zajezierce had the smallest. The observed differences between these populations were statistically significant. In most cases, the *G. robertianum* plants of the railway track populations had a higher number of leaves in rosettes than the forest ones. The observed difference between the railway track and forest populations was statistically significant (Fig. 7A, B). A greater number of leaves in plants of the railway track populations is a feature that differentiates the track and forest populations and that can be adaptive to the occurrence of *G. robertianum* on railway tracks.

As for the average fresh mass of the *G. robertianum* rosettes from the studied railway track and forest populations, plants of the track populations from Białystok Fabryczny and Waliły-Station (2008) had the heaviest rosettes and plants of the forest population from Gródek had the lightest. The observed differences were statistically significant. In most cases, greater weight of rosettes was characteristic of the *G. robertianum* plants of the railway track populations rather than forest populations. The difference in weight of rosettes between plants of the railway track and forest populations was statistically significant (Fig. 7C, D).

It was shown that plants from the railway track and forest populations after spraying with the herbicide contained different concentrations of glyphosate and AMPA in their tissues, despite the same dose of the herbicide used for spraying. The higher average glyphosate and AMPA contents were found in plants from the railway track populations than in the forest ones. Most likely, the content of glyphosate and AMPA in plants after spraying with the herbicide depends on the number of leaves in rosettes and their distribution on a plant, i.e., the surface that has been in direct contact with the herbicide. Plants of the railway track populations were characterized by a higher number of leaves in rosettes and higher fresh weight of overground parts than plants of the forest populations. Therefore, plants of the railway track populations took a higher dose of the herbicide.

## Discussion

Within this study, it was examined whether the use of the herbicide on railway tracks could have contributed to an increase in tolerance to the glyphosate in the *G. robertianum* plants of the railway track populations compared to plants of the forest populations.

### Effect of glyphosate on *G. robertianum* plants

It is known from the literature that most plants after spraying with glyphosate absorb it very quickly. Once inside the plants, glyphosate along with photosynthesis products gets through phloem, and to a lesser extent through xylem. The effect of glyphosate on sensitive plants is manifested by inhibited plant growth, chlorosis and necrosis, yellowing of leaves and shoots, followed by death of entire overground parts of plants and their roots after about 3 weeks. If the plant is not completely destroyed by glyphosate, the regrowing leaves are distorted (Campbell et al. 1976; Vaughn and Duke 1986; Eker et al. 2006; Tuffi Santos et al. 2009; Adamczewski et al. 2011; Adamczewski 2014; Safety data sheet Roundup 360 SL 2009). No such harmful effect of glyphosate was observed in *G. robertianum* plants from railway tracks, but it was visible in plants from forests.

On the leaves of the *G. robertianum* plants from the railway track populations, chlorosis after the herbicide spraying was initially observed, but gradually it began to disappear. This indicates gradual regeneration of the leaves. No such a phenomenon was observed in plants from the forest populations of *G. robertianum*, where the leaves began to die after a month. The appearance of chlorosis on leaves can be explained by reference to literature. The reduction of the chlorophyll content in leaves or strong chlorosis of the youngest leaves in different plant species, after applying glyphosate was found for example by Kitchen et al. (1981) and Eker et al. (2006). The formation of chlorosis on leaves after applying glyphosate can be explained by the degeneration of chloroplasts (Campbell et al. 1976) or/and inhibition of chlorophyll formation (Cole et al. 1983). Glyphosate and AMPA reduce photosynthesis in plants, but through various mechanisms: glyphosate increases chlorophyll degradation, while AMPA disrupts chlorophyll biosynthesis. Both the increased degradation of chlorophyll and its reduced biosynthesis cause yellowing of leaves and the appearance of necrosis on them (Gomes et al. 2016; Van Bruggen et al. 2018). However, if a plant shows some glyphosate tolerance, the initial chlorosis on leaves may disappear over time, as was seen with the *G. robertianum* plants of the railway track populations. For example, in the work by Tuffi Santos et al. (2009) two species of eucalyptus, treated with the glyphosate were studied. The effect of glyphosate (chlorosis, curling, wilting or complete withering of leaves) was the stronger the higher the dose was used. When a lower dose of herbicide was used, complete regeneration of the tested plants was observed over time (Tuffi Santos et al. 2009). That is, as in the case of the *G. robertianum* plants from the railway track populations, but here a high dose of herbicide was used, and yet in plants from the railway track populations’ gradual disappearance of chlorosis on the leaves was observed. This result also indicates the tolerance of the *G. robertianum* plants from the railway track populations to glyphosate.

In the *G. robertianum* plants from the railway track populations after the herbicide spraying, no altered leaf cells’ ultrastructure was observed compared to the controls. Numerous electron-dense deposits were observed only in vacuoles. In contrast, plants from the forest population had clearly visible damage to cells and organelles ultrastructure, especially chloroplasts. For comparison, in Vaughn and Duke (1986), after the use of glyphosate in the *Glycine max* (L.) Merr., disturbances in the structure of gran thylakoids and the arrangement of thylakoids in arcuate/spiral-shaped grana was observed. In Campbell et al. (1976), in glyphosate-sensitive *Agropyron repens* L., in cells’ ultrastructure after herbicide using was observed: swelling of chloroplasts, their aggregation, disturbed orientation of gran polarity, complete destruction of chloroplasts, slight swelling of other organelles (mitochondria, endoplasmic reticulum), around the plasmalemma and cell wall electron-dense bodies occurred. In turn, in Tuffi Santos et al. (2009) research in cells’ ultrastructure of leaves in eucalyptuses, was observed: plasmolysis, sponge mesophyll hypertrophy (excessive cells’ growth), hyperplasia (increase in cells’ number), necrosis in the upper epidermis, palisade mesophyll cells with dark content, thinning of cell walls, necrosis of cells forming a conductive bundle and the appearance of cells with dark content nearby conductive bundles, as well as large intercellular spaces in mesophyll, thinning of the palisade and spongy mesophyll cells. Such intensified changes in leaf cells’ ultrastructure like in above cited research, were observed only in the *G. robertianum* plants of the forest population, while they were not observed in plants of the railway track populations, which confirms their increased tolerance to the glyphosate.

The lack of tolerance to the glyphosate in the *G. robertianum* plants of the forest populations shown in this study agrees with the observations made by Gove et al. (2007), who found that the *G. robertianum* forest population was one of the most sensitive to glyphosate spray among the six species studied. After spraying, plants’ biomass decreased significantly compared to the control, the more the higher the glyphosate dose was used. The response to glyphosate in *G. robertianum* from natural habitats was also studied by Strandberg et al. (2012) and Boutin et al. (2014) in pot experiments. The glyphosate was applied at the 6 to 10 leaf (vegetative phase) stage and at the bud stage (reproductive phase). This research showed that the least sensitive from studied species was *G. robertianum* at the early as well as the late growth stages. It was also shown that *G. robertianum* was the most tolerant species at the reproductive stage (Strandberg et al. 2012; Boutin et al. 2014), which is consistent with the observations of Gove et al. (2007), where was not found that spraying suppressed the *G. robertianum* flowering; in comparison with other forest species, these plants proved to be the least sensitive. The above examples show that the response of *G. robertianum* from natural habitat to glyphosate is quite varied and may depend, inter alia, on from the tested plant phenological stages.

The better condition of both whole plants and the youngest leaves in the railway track populations compared to plants of the forest populations was observed. Also, fewer plants of *G. robertianum* from railway tracks withered after the herbicide treatment than plants from forest habitats. Therefore, the plants of *G. robertianum* from the railway tracks had a higher tolerance to glyphosate than plants from the forests. While the response to glyphosate was quite similar in the forest populations (in contrast to studies by: Gove et al. 2007; Strandberg et al. 2012; Boutin et al. 2014, where some differences between natural populations was observed), the herbicide response in plants from the track populations was quite variable. The observed differences in the herbicide tolerance of plants within the railway track populations could have resulted from differences in intensity of the herbicide spraying at individual stations. Waliły-Station is small, while Białystok Fabryczny and Zubki Białostockie are large stations, so the herbicide spraying could be more intense there. This could have resulted in the higher herbicide tolerance of plants from the larger train stations. However, further research is still needed to confirm this.

#### Plant tolerance to glyphosate

It is also worth considering what we understand by the concepts of tolerance and resistance to herbicides and how common these phenomena are in the case of glyphosate. By tolerance to a herbicide we mean the ability of a plant species to survive and reproduce after using the recommended dose of a herbicide. Tolerance is a hereditary feature, passed on to offspring (Adamczewski 2014; Rao 2014). Within populations of the same plant species, herbicides can affect the selection process, which involves in weeds getting resistant to herbicides that are often and widely used (Christoffers 1999; Adamczewski and Dobrzański 2012b). In turn, by weed resistance to herbicides we mean the natural or induced ability of some plants to survive and reproduce after exposure to a lethal dose of a herbicide, while the rest of the population dies. The main factors influencing the development of tolerance or resistance among plants (weeds) are: use of herbicides with one mechanism of action in the same area for several years, improper selection of agents for the weed species composition, inadequate plant development phase at the time of application, adverse weather and environmental conditions for the proper operation of the herbicide (Krysiak et al. 2011; Adamczewski and Dobrzański 2012a; Adamczewski 2014; Rao 2014; EPPO Standard PP 1/213(4) 2015; Buddenhagen et al. 2019). The dose of the herbicide may also influence the development of tolerance or even resistance to the herbicide. It is presumed that high doses of the herbicide affect one resistance gene at the site of action (monogenic resistance). Conversely, lower doses (or less effective herbicides) contribute to multi-gene resistance (polygenic resistance), which increases metabolism. In theory, high doses of herbicides and highly active herbicides will control almost all sensitive plants, leaving only very resistant individuals associated with resistance at the site of action, which is usually monogeneously inherited. In turn, selection based on polygenic resistance depends on genetic recombination involving several genes throughout the genotype. In the case of weed resistance to herbicides, it cannot be ruled out that in many situations the resistance of plants to herbicides may be arise due to epigenetic changes (Devine and Preston 2000; Adamczewski 2014). Thus, the dose applied and the amount of herbicide uptake by the plants will also be of importance in the development of resistance.

Another issue raised in this paper was to answer the question what actual dose of the herbicide the *G. robertianum* plants receive during spraying. It was shown that higher average glyphosate and AMPA content was found in *G. robertianum* plants of the railway track populations than forest ones, in spite of the same dose of the herbicide used for spraying. The obtained data indicate that the content of glyphosate and AMPA in plants after spraying with the herbicide depends on a number of leaves in rosettes and their distribution on a plant, i.e., the surface that was in direct contact with the herbicide (higher number of leaves and higher fresh mass of overground parts was found in plants from railway tracks). The more leaves a plant has, the greater the total surface area of leaf blades is. It is known from the literature data that foliar absorption of pesticides is a complex process depending, among others, on characteristics of leaf surface in sprayed plants (Wang and Liu 2007). For example, in the studies by Liu (2003) and Wang and Liu (2007) it was shown that effectiveness of glyphosate uptake by grasses does not depend on a dose used for spraying, but does on a dose accumulated on the leaf surface. These works would confirm the thesis that the dose of the herbicide taken by the *G. robertianum* plants could have depended on the total leaf area in the plants. The works of other researchers show that such factors as working conditions of the sprayer, physical properties of the spray mixture, small structures on the surface of leaves and the dominant microclimate can have an influence on the effectiveness and efficiency of the pesticide use (Reichard et al. 1986; Fox et al. 1992; Yu et al. 2009). It is possible that the coverage of the *G. robertianum* plants with glandular trichomes, of different sizes (Pedro et al. 1990) could also have had an impact on the uptake of the herbicide by these plants. The influence of various structures on the leaf surface on the glyphosate uptake were studied by many researchers, for example, Chachalis et al. (2001a, 2001b), Hatterman-Valenti et al. (2006). In the work by Yu et al. (2009) was shown that evaporation of solutions from the leaf surface in *Pelargonium* was longer for leaves covered with wax (*P. stenopetalum*) and shorter for leaves densely covered with trichomes (*P. tomentosum*). Different length of trichomes in *G. robertianum* can contribute to the differentiated retention of the herbicide drops, away from the leaves surface, and protect it against the herbicide.

Developing tolerance or even resistance to glyphosate is associated with its constant use. In areas where it is used, biotypes form that show resistance to glyphosate (Steinmann et al. 2012; Adamczewski 2014). The list of HRAC (Herbicide Resistance Action Committee) indicates 48 weed species resistant to glyphosate, confirmed in about 20 countries, including one in Poland (Adamczewski 2014; Heap 2020). In the USA, as a result of the increasing use of glyphosate, weeds have appeared in the fields that are resistant to this active substance, for example, *Conyza canadensis* (Adamczewski and Dobrzański 2012a).

Regular spraying on railway tracks has caused that the *G. robertianum* populations occurring there, including the population from Waliły-Station described by Wierzbicka et al. (2014), not only adapted to excessive insolation and water shortage, but also to spraying with herbicides. Until now, few plant biotypes have been found on railway tracks that tolerate or become resistant to glyphosate used there. These are such biotypes from railway tracks as: *C. canadensis* in Poland, the Czech Republic, Spain and Japan (Chodová et al. 2009; Adamczewski et al. 2011; Hamouzová et al. 2009, 2012; Adamczewski 2014; Nagai et al. 2015; Amaro-Blanco et al. 2019; Heap 2020), *Conyza bonariensis* in Spain (Amaro-Blanco et al. 2019), *Lolium rigidum* in Australia (Malone et al. 2012; Heap 2020), *Brassica napus* in Switzerland (Schoenenberger and D’Andrea 2012) and *Parthenium hysterophorus* in the USA (Fernandez et al. 2015; Heap 2020). In Poland, individuals of *C. canadensis* found on railway tracks in the suburbs of the Poznań city, where the glyphosate is constantly used, were the resistant biotype. The mechanism of resistance of this biotype to glyphosate has not yet been known (Adamczewski et al. 2011; Adamczewski 2014; Heap 2020). Demonstration of the increased glyphosate tolerance in the *G. robertianum* plants of the railway track populations relative to the forest populations is the first case described for this species, and the second one in Poland for the species found on railway tracks.

Increased tolerance to the glyphosate in the *G. robertianum* plants from the railway track populations, compared to plants from the forest populations indicates the occurrence of microevolutionary processes due to the action of the selection factor which is the use of herbicides on railway tracks. Microevolutionary processes resulting from environmental pollution over time can lead to the formation of new forms, varieties or even subspecies (Bone and Farres 2001; Hendry and Kinnison 2001; Ashley et al. 2003; Medina et al. 2007; Wójcik et al. 2013; Wójcik and Tukiendorf 2014; Bemowska-Kałabun et al. 2020a, 2020b; Hendry 2020; Wierzbicka et al. 2020). It is worth adding that, the study of microevolutionary processes occurring in anthropogenically changed areas (e.g., railway tracks) is now one of the basic issues of ecotoxicology (Bickham 2011; Coutellec and Barata 2011; Vandegehuchte and Janssen 2011; Van Straalen et al. 2011).

### Mechanisms of plant tolerance to glyphosate

Finally, it is necessary to consider what mechanism may have contributed to the increased glyphosate tolerance in the *G. robertianum* plants of the railway track populations. The mechanism of the glyphosate action is inhibiting the enzyme, 5-enolpyruvylshikimate-3-phosphate (EPSP) synthase. It is a key enzyme of the shikimic acid pathway, found in plants, fungi and microorganisms. Its inhibition results in the lack of synthesis of essential aromatic amino acids (tryptophan, phenylalanine, tyrosine). This leads to disruption of protein synthesis and cell death (Kishore et al. 1992; Schuette 1998; Blackburn and Boutin 2003; Rojano-Delgado et al. 2012). Glyphosate and its main breakdown product, AMPA inhibit the activity of antioxidant enzymes and induce the accumulation of reactive oxygen species (so-called ROS), which in turn induce physiological dysfunction and damage of cells (Gomes et al. 2016; Van Bruggen et al. 2018). Plants treated with glyphosate do not produce secondary aromatic compounds, including antimicrobial phytoalexins, which protect plants from pathogens. Consequently, glyphosate-treated plants often die due to infection with root pathogens that are commonly found in soil. Even sub-lethal glyphosate concentrations in plants, for example derived from residues present in soil or water, reduce plant resistance to pathogens. In addition to reduced plant resistance, indirect effects of glyphosate and AMPA can also affect plant health, such as changes in the rhizosphere microbiome and endophytic microbiome (Rosenbaum et al. 2014; Van Bruggen et al. 2018). Another mechanism of the glyphosate action is desiccation, that is dehydration of plant tissues. Closing stomata reduces respiration, causing interference in photosynthesis (Blackburn and Boutin 2003; Parreira et al. 2015).

One of the mechanisms of plant resistance to glyphosate is associated with mutation of the EPSPS (Pro106) sequence or may be the result of EPSPS gene extension. For example, studies on the resistant *Amaranthus palmeri* biotype have shown that the plant is overexpressed by the EPSPS gene due to extension of the EPSPS gene (Gaines et al. 2010; Powles and Yu 2010; Adamczewski 2014; Rao 2014). Research by Yuan et al. (2002) on *Dicliptera chinensis* is an example of the glyphosate resistance resulting from the higher EPSP synthase activity. In turn, studies conducted on the resistant *Lolium multiflorum* strain prove that resistance to glyphosate may also result from its limited transport in the plant. In control, glyphosate was transported to the base of the leaf blade, stem and roots, and in a strain resistant to glyphosate, it was stored at the end of the leaf blade. In this case, glyphosate was moving in xylem and its penetration into the apoplast was limited (Perez-Jones et al. 2007). The glyphosate tolerance/resistance of plants, associated with reduced glyphosate concentration in meristematic tissues due to impairment or limited uptake and translocation of the herbicide, is also described, for example, by Feng et al. (2004) and Wakelin et al. (2004). Plant resistance to glyphosate may also be associated with the herbicide sequestration in cellular compartments such as vacuoles (Ge et al. 2010). Probably, the last mechanism may be responsible for the increased tolerance in the *G. robertianum* plants of the railway track populations. This would be indicated by the increased content of electron-dense deposits in vacuoles of leaf cells in plants from the railway track populations compared to the control. The fact that in addition to glyphosate AMPA was also found in plant tissues may indicate ability of the *G. robertianum* plants to metabolize glyphosate. For example, in the work by Rojano-Delgado et al. (2012) it was shown that *Mucuna pruriens* is highly tolerant to glyphosate and is capable of metabolizing glyphosate, as its degradation products, including AMPA were found in its tissues. In turn, in the work of Takano et al. (2019) in a glyphosate-resistant and biosensitive biotype *Bidens subalternans*, similar contents were found in both glyphosate and AMPA after glyphosate treatment, and it was found that differential metabolism does not explain glyphosate resistance in *Bidens subalternans*. It is also worth recalling the possibility discussed earlier that the trichomes on the *G. robertianum* leaves can provide protection against reaching the herbicide leaf surface. However, to confirm the listed mechanisms explaining the increased tolerance of *G. robertianum* from the railway track populations to the glyphosate and to exclude other possible mechanisms further testing should be carried out.

## Conclusion

In this study, tolerance to the glyphosate in *G. robertianum* was found to be higher in the railway track plants than in the forest populations. The observed differences in the herbicide tolerance between the *G. robertianum* plants from the railway track and forest populations are an excellent example of the occurrence of microevolutionary processes on railway tracks. The glyphosate tolerance in the *G. robertianum* railway track populations explains the mass occurrence of this species on railway tracks in north-eastern Poland. Demonstration of the increased glyphosate tolerance in the *G. robertianum* plants of the railway track populations is the first case described for this species, and the second one in Poland for the species found on railway tracks.
